# A Study of Tpeak-Tend/QT Interval Ratio in Predicting Heart Failure in ST-Elevation Myocardial Infarction and Its Correlation With N-terminal Pro B-type Natriuretic Peptide (NT-proBNP)

**DOI:** 10.7759/cureus.68495

**Published:** 2024-09-02

**Authors:** Sharan Badiger, Venugopal Hanumappa, Avinash V Jugati

**Affiliations:** 1 General Medicine, Shri B. M. Patil Medical College Hospital and Research Centre, Bijapur Lingayat District Education (Deemed to be University), Vijayapura, IND

**Keywords:** nt-probnp, qt interval, major adverse cardiac events, acute myocardial infarction, tpeak-tend, heart failure

## Abstract

Background

Acute ST-elevation myocardial infarction (STEMI) is the most common cause of mortality across the world. The electrocardiogram (ECG) Tpeak-Tend/QT interval appears to be a measure of the left ventricle's transmural dispersion of repolarization (TDR). Prolongation of this time could indicate adverse cardiac events like heart failure, arrhythmias, etc. Heart failure is a clinical syndrome that includes signs and symptoms such as peripheral edema and high jugular vein pressure. N-terminal pro B-type natriuretic peptide (NT-proBNP) is an effective predictor of left ventricular function and also helps in predicting the disease prognosis. Elevated levels of NT-proBNP are seen in many cases of left ventricular dysfunction. This study aims to evaluate the predictive utility of the Tpeak-Tend/QT interval ratio in predicting major adverse cardiac events (MACEs), such as heart failure, by examining the Tend/QT interval ratio values with NT-proBNP in STEMI.

Methodology

This cross-sectional study was conducted at a tertiary hospital between April 2024 and June 2024. It included STEMI patients, excluding those with non-STEMI (NSTEMI), valvular heart diseases, bundle branch block, or pacemakers. The patients with a Tpeak-Tend/QT ratio < 0.3 were included in group A, and the Tpeak-Tend/QT ratio > 0.3 in group B. They were monitored for MACE-like heart failure during hospitalization and were compared with a study of the Tpeak-Tend/QT interval ratio in predicting heart failure in STEMI and its correlation with NT-proBNP levels.

Results

In this study, out of 45 patients, male predominance was observed, with 35 (78%) being men and 10 (22%) being women. In group A, the most common age group was 60-70 years, with 16 (51%) patients; in Group B, it was 50-60 years, with six (42.8%) patients. Out of 31 patients in group A, 25 were male, and six were female. In group B, out of 14 patients, 10 were male, and four were female. In this study, out of the 45 patients included, 12 (85%) among 14 patients who had MACEs like heart failure had a Tpeak-Tend/QT interval ratio of more than 0.3, and their measured NT-proBNP levels were also more than 900 pg/mL, thus showing a statistically significant association between Tpeak-Tend/QT interval ratio and NT-proBNP.

Conclusion

The present study showed an increased Tpeak-Tend/QT interval ratio and NT-proBNP in patients who developed heart failure in STEMI. As ECG is a readily available and affordable tool, the Tpeak-Tend/QT interval ratio can be used along with conventional markers like NT-proBNP to predict MACE in patients with STEMI.

## Introduction

Acute ST-elevation myocardial infarction (MI) is one of the conditions with increased mortality and morbidity throughout the world. The prevalence of STEMI has increased mortality even with advancements in treatment. The majority of the time, failure of left ventricular ejection, a consequence of abrupt coronary occlusion, is the reason for death. It is shown that the dispersion of transmural repolarization might be measured using the Tpeak-Tend interval. The QT interval rises linearly with increased heart rate and body weight when the Tpeak-Tend interval increases concurrently, which is associated with various MACE [1]. The Tpeak-Tend/QT interval ratio has been proposed as a more precise indicator of heart failure. The Tpeak-Tend/QT interval ratio remains consistent within the restricted interval of 0.25 to 0.3. Increased Tpeak-Tend/QT interval ratios have been associated with various MACE, including heart failure [2].

The ventricular myocardium produces and secretes cardiac neuro-hormone, N-terminal fragment, and B-type natriuretic peptide (BNP). It is commonly recognized that a rise in left ventricular wall tension triggers their release. It is well established that individuals with acute coronary syndromes (ACS) have higher levels of these markers [3]. In this case, ventricular dysfunction and myocardial ischemia may cause an increase in cardiac N-terminal pro B-type natriuretic peptide (NT-proBNP) expression, which may be followed by enhanced secretion [4]. Furthermore, it has been demonstrated that following an MI, NT-proBNP levels are predictive of heart failure and death. Patients with NT-proBNP higher than 900 pg/mL are highly likely to have heart failure [5].

## Materials and methods

This cross-sectional study was conducted at Shri B. M. Patil Medical College Hospital and Research Centre, BLDE (Deemed to be University), Vijayapura, between April 2024 and June 2024. The institutional ethical clearance was obtained (IEC/1093/2023-24). The sample size was calculated with a 95% level of confidence interval and precision of 10%. The inclusion criteria were all patients admitted with STEMI, and the exclusion criteria were patients with NSTEMI, bundle branch block, and valvular heart disease.

For all patients who were admitted, a detailed clinical history, physical examination, electrocardiogram (ECG), laboratory investigations like troponin I, creatine phosphokinase-MB (CPK-MB), complete blood count, random blood sugar, lipid profile, and renal profile were done. NT-proBNP values were measured by using an enzyme-linked fluorescence assay (ELFA) [6]. Other relevant investigations were conducted, such as echocardiography and coronary angiography (if required). Of the 52 patients enrolled, 45 patients who fulfilled the inclusion criteria were included in the study, and seven patients were excluded based on the exclusion criteria (NSTEMI = four; bundle branch block = two; valvular heart disease = one). The Tpeak-Tend/QT interval ratio was calculated using a 12-lead ECG, and the QT interval was measured from the start of the QRS complex to the end of the T wave [7]. Using Bazett's formula, the corrected QT interval (QTc) was determined [8]. The Tpeak-Tend interval is the point when the iso-electric baseline is crossed by the tangent of the greatest downslope of the T wave's descending limb. This interval was measured using the “tangent” method in ST-segment raised leads from the peak of the T wave to the end of the T wave (Tend) [9]. The maximum deflection of the T wave is known as the Tpeak. Patients with a Tpeak-Tend/QT interval ratio < 0.3 were grouped as group A, and >0.3 were grouped as group B. The patients were observed for the development of heart failure based on Killip Classification criteria during their in-hospital stay [10]. Further, the correlation between the severity of heart failure and NT-proBNP levels was studied.

The statistical analysis was done using Statistical Package for Social Science (version 20) software (IBM Corp, Armonk, NY). The data were represented using graphs, counts and percentages, mean and standard deviation, continuous variables, and those that are regularly distributed were compared using an independent t-test; for categorical variables, a chi-square test was used. Mann-Whitney U test was used for variables that were not regularly distributed. All statistical tests were performed in two-tailed, and the results were considered statistically significant if the p-value was <0.05.

## Results

Based on the Tpeak-Tend/QT interval ratio, 45 patients in total were included in the study and split into two groups: A (<0.3) and B (>0.3). The typical age group in group A was from 60-70 years (n = 10), with eight (80%) patients being male and two (20%) being female. The typical age in group B was 50-60 years (n = 6), with four (66%) patients being male and two (33%) being female. An overview of demographical and clinical data is summarized in Table 1. The hemodynamic and laboratory data of all patients are depicted in Table 2.

**Table 1:** Demographical and clinical data

Demographical data	Group A (n = 31)		Group B (n = 14)		p-value	Chi-square
Age group (in years)	Male (%)	Female (%)	Male (%)	Female (%)		
21-30	0 (0)	0 (0)	1 (7.14)	0 (0)	0.89	8.74
31-40	3 (9.6)	0 (0)	1 (7.14)	0 (0)		
41-50	3 (9.6)	1 (3.2)	1 (7.14)	0 (0)		
51-60	5 (16.1)	2 (6.2)	4 (28.5)	2 (14.2)		
61-70	8 (25.8)	2 (6.2)	2 (14.2)	1 (7.14)		
71-80	7 (22.5)	1 (3.2)	1 (7.14)	1 (7.14)		
Clinical data	Group A (n = 31)		Group B (n = 14)		p-value	Chi-square
	Male (%)	Female (%)	Male (%)	Female (%)		
Gender	25 (80%)	6 (20%)	10 (71%)	4 (29%)	0.66	46.8
Diabetes	2 (6.25)	0 (0)	4 (28.5)	2 (14.2)	0.78	48.2
Hypertension	4 (12.9)	2 (6.4)	8 (57.1)	3 (21.4)	0.26	17.2
Smoking	11 (34.3)	0 (0)	11 (78)	0 (0)	0.85	56.3
Alcohol	6 (19.3)	0 (0)	5 (35.7)	0 (0)	0.52	35.6
Chest pain	20 (64)	7 (22.5)	9 (64)	3 (21.4)	0.62	44.8
Dyspnea	16 (51)	6 (19.3)	6 (42.8)	2 (14.2)	0.35	19.2
Palpitation	8 (25.8)	4 (12.9)	4 (28.5)	1 (14.8)	0.25	17.7

**Table 2:** Hemodynamic and laboratory data *p < 0.05, statistically significant

Hemodynamic and laboratory data	Group A (n = 31)		Group B (n = 14)		Normal range	p-value	Chi-square
	Mean	SD	Mean	SD			
Pulse rate (beats per minute)	86.7	27.3	87.4	19.1	60-100 (beats per minute)	0.84	52.3
Systolic blood pressure (mmHg)	110.5	30.3	108.2	25.9	100-130 (mmHg)	0.75	47.5
Hemoglobin (gm%)	13.3	2.4	13.4	2.5	12-15 g/dL	0.48	26.8
White blood cells (10^3^/L)	12,236.9	3,493.6	1,2256.8	3,505.5	4,000-10,000 10^3^/L	0.15	14.6
Troponin I (ng/dL)	5,449	7911	9888	58.41	<19 ng/dL	0.21	35.6
Creatinine (mg/dL)	1.12	0.38	0.89	0.3	0.4-1.1 mg/dL	0.09	17.3
Random blood sugar (mg/dL)	160	82	180	88	70-140 mg/dL	0.37	27.8
Total cholesterol (mg/dL)	159	40	189	73	<200 mg/dL	0.98	34.6
Triglycerides (mg/dL)	140	63	179	75	<150 mg/dL	0.89	47.8
Left ventricular ejection fraction < 40%	2 (6.3%)		35 (74.5%)		40-60%	0.05*	17.2

All 45 patients were grouped into A and B based on the Tpeak-Tend/QT interval ratio. Patients with a Tpeak-Tend/QT interval ratio < 0.3 in group A (n = 31) and a Tpeak-Tend/QT interval ratio > 0.3 in group B (n = 14). Out of 31 patients in group A, 25 (80%) were male, and 10 (20%) were female. Group B included 14 patients, of which six (71%) were male and four (29%) were female (Table 3). There was statistical significance among male patients in both group A and group B patients.

**Table 3:** Distribution of Tpeak-Tend/QT interval ratio *p < 0.05, statistically significant

Gender	Group A	Group B	p-value	Chi-square
	n (%)	n (%)		
Male	25 (80)	10 (71)	0.05*	16.01
Female	6 (20)	4 (29)	0.06	17.50

A 2D echo-doppler investigation was used to examine the left ventricle ejection fraction. Of the 31 patients in group A, three patients (9.6%) had heart failure with LVEF <40%. Of 14 patients in Group B, 12 patients (85.7%) had heart failure with LVEF <40%. In Group A, 28 of 31 (90.3%) patients had heart failure with LVEF >40%. In Group B, two out of 14 patients (14.3%) had heart failure with LVEF >40%. The estimated NT-proBNP value of <900 pg/mL was seen in 25 of 31 (80%) patients in group A, two of 14 patients (14.3%) in group B, and NT-proBNP of >900 pg/mL was seen in six out of 31 (19%) patients in Group A and 12 of 14 (85.7%) patients in Group B (Table 4).

**Table 4:** Comparison of major adverse cardiac events with NT-proBNP levels *p < 0.05, statistically significant NT-proBNP, N-terminal pro B-type natriuretic peptide

Major adverse cardiac event	Group A (n = 31) (%)	Group B (n = 14) (%)	Chi-square	p-value
Heart failure (LVEF <40%)	3 (9.6%)	12 (85.7%)	36.61	*0.01
Heart Failure (LVEF >40%)	28 (90%)	2 (14.3%)		
NT-proBNP levels	Group A (n = 31) (%)	Group B (n = 14) (%)	Chi-square	p-value
NT-proBNP (<900 pg/mL)	25 (80%)	2 (14.3%)	56.52	*0.01
NT-proBNP (>900 pg/mL)	6 (19%)	12 (85.7%)		

## Discussion

This study aimed to determine whether the Tpeak-Tend/QT interval ratio interval might be used to predict serious adverse cardiac events, such as heart failure, and compare it with NT-proBNP, which occurs in patients with STEMI while hospitalized. We also evaluated the risk variables associated with STEMI. This study included 45 acute MI patients in total. Thirty-one of the 45 patients were in group A if their Tpeak-Tend/QT interval ratio was less than 0.3, while group B consisted of 14 patients if their interval was more significant than 0.3.

In this study, the most prevalent age group was 50-70. Similarly, the mean age of 353 patients who participated in the survey by Panikkath et al. in 2011 was between 50 and 70 years old [11]. Our findings are consistent with similar studies and suggest that aging is a significant non-modifiable risk factor for MI development. The study conducted by Zumhagen et al. in 2016 included 178 patients, of whom 124 (70%) were male. This indicates that there was a male predominance in the current study, with 35 (78%) patients being male and 10 patients (22%) being female [12]. In another study conducted in 2020 by Kazemi B et al., it was observed that 138 (73.96%) of the 188 cases were males, and 50 of 188 cases (26.03%) were females [13]. This indicates that MI development is more common in males. Left ventricular ejection fraction (LVEF) aids in evaluating the heart's systolic function. LVEF is a noteworthy indicator of cardiac mortality. In our study, 12 of 14 (85%) patients in group B who had LVEF less than 40% had a Tpeak-Tend/QT interval ratio larger than 0.3. The Tpeak-Tend/QT interval ratio and LVEF of patients with an older MI were found to be adversely connected in a study conducted by Mugnai et al. in 2016 [14]. The results also showed that the Tpeak-end/QT interval ratio is higher than 0.3 when the LVEF is less than 40%.

This study mainly compared the Tpeak-Tend/QT interval ratio and NT-proBNP in predicting adverse cardiac events like heart failure. The results demonstrated that in STEMI patients, a Tpeak-Tend/QT interval ratio higher than 0.3 was associated with an increased incidence of MACE. Heart failure is the main adverse cardiac event (group A = 3 (9.6%), group B = 12 (85.7%)). The findings of this study are consistent with another study done in 2019 by Alhamaydeh et al., which revealed that the Tpeak-Tend/QT interval ratio is a very strong indicator of death in patients suffering from acute MI [15]. Results were significantly worse when the Tpeak-Tend/QT interval ratio was more significant than 0.3 when analyzing various factors. In particular, heart failure affected 85% of patients with a ratio above 0.3, and 78% of patients had an ejection fraction below 40%. These rates were significantly greater than those found in patients whose T-peak-end/QT interval ratio was less than 0.3. In our study, 12 (85%) of the 14 patients with heart failure during hospital stay had NT-proBNP of more than 900 pg/mL and a Tpeak-Tend/QT interval ratio of more than 0.3. In a study by Rørth et al., NT-proBNP levels in heart failure and decreased ejection fraction seem to be higher [16]. These results are crucial for comparing the lower ejection fraction and natriuretic peptide concentrations in heart failure throughout our studies.

This study demonstrated that a simple bedside ECG parameter like Tpeak-Tend/QT interval along with NT-proBNP was significantly high in patients with MACEs like heart failure. Using data from 45 patients who had STEMI, we analyzed the data. The results showed that the Tpeak-Tend/QT interval ratio is an excellent predictor of MACEs in STEMI, which can help reduce these patients' high death and morbidity rates by providing early intervention.

Limitation

This was a single-center study with limited sample size; future multi-centric studies with larger sample sizes will help improve the Tpeak-Tend/QT interval ratio-based MACE prediction accuracy in STEMI patients. Expanding the sample size and extending the follow-up period would be advantageous to fully understand the relationship between MACE, such as heart failure, and the Tpeak-Tend/QT interval ratio.

## Conclusions

The present study indicated that patients with STEMI who had elevated NT-proBNP levels and an ECG with a Tpeak-end/QT interval ratio larger than 0.3 were at more risk of major adverse cardiac events, such as heart failure. The ratio is determined using the first ECG, as it is a simple, less expensive diagnostic investigation than NT-proBNP, which is more expensive and less accurate in predicting heart failure. Therefore, the Tpeak-Tend/QT interval ratio can be used to predict major adverse cardiac events like heart failure.
